# Digital Intelligence, Emotional Intelligence, Self-Disclosure, and Fear of Missing Out Among Thai Generation Z: A Cross-Sectional PLS-SEM Study

**DOI:** 10.3390/ijerph23080948

**Published:** 2026-07-23

**Authors:** Naphat Wuttaphan, Hisako Matsuo, Bovornpot Choompunuch

**Affiliations:** 1 Department of Human Resource and Organization Management, Faculty of Management Science, Pibulsongkram Rajabhat University, Phitsanulok 65000, Thailand; naphat.w@psru.ac.th; 2 Department of Sociology and Anthropology, College of Arts and Sciences, Saint Louis University, St. Louis, MO 63108, USA; hisako.matsuo@slu.edu; 3 Department of Educational Psychology and Guidance, Faculty of Education, Mahasarakham University, Sarakham 44000, Thailand; 4 Research Unit of Interdisciplinary and Lifelong Learning, Faculty of Education, Mahasarakham University, Sarakham 44000, Thailand

**Keywords:** digital intelligence, emotional intelligence, fear of missing out, Generation Z, self-disclosure, social media, Thailand

## Abstract

**Highlights:**

**Public health relevance—How does this work relate to a public health issue?**

- FOMO reflects a growing digital mental health concern among young people who are constantly exposed to online social comparison.
- Understanding psychological and digital factors associated with FOMO can support healthier social media use among Generation Z.

**Public health significance—Why is this work of significance to public health?**

- The study provides evidence from Thailand, a Southeast Asian context that remains underrepresented in digital wellbeing research.
- The findings suggest that digital competence alone may not be sufficient to protect young people from FOMO-related risks.

**Public health implications—What are the key implications or messages for practitioners, policy makers and/or researchers in public health?**

- At this stage, the findings should be treated as preliminary and should not be used on their own to guide digital-wellbeing programs or public health policy.
- Future studies should refine the measurement model and confirm which dimensions of digital intelligence are related to FOMO before translating the findings into practice.

**Abstract:**

Fear of missing out (FOMO) has become an important digital mental health concern among Generation Z, a population highly engaged with social media and online interaction. This study examined the associations among digital intelligence, emotional intelligence, self-disclosure, and FOMO among Generation Z individuals in Thailand. A cross-sectional quantitative study was conducted from December 2025 to January 2026. Data were collected from 322 participants using self-administered questionnaires and analyzed using descriptive statistics and partial least squares structural equation modeling. The results showed that digital intelligence was positively associated with FOMO, whereas emotional intelligence and self-disclosure were not significantly associated with FOMO. Self-disclosure did not mediate the associations between emotional intelligence, digital intelligence, and FOMO. However, the measurement model showed acceptable internal consistency but limited convergent validity, as average variance extracted values were below the conventional threshold for all constructs. Therefore, the structural findings should be interpreted as preliminary and exploratory. The positive association between digital intelligence and FOMO may reflect greater exposure to online social activity, social comparison, or possible overlap between the constructs, rather than a harmful effect of digital intelligence itself. These findings contribute initial evidence from Thailand but should not be used alone to guide public health practice or policy. Future studies should refine the measurement instruments, examine individual dimensions of digital intelligence, and confirm the observed relationships using stronger psychometric and longitudinal designs.

## 1. Introduction

Social media has become deeply embedded in the everyday lives of young people, shaping how they communicate, build relationships, express identity, seek information, and compare themselves with others [1]. For Generation Z, who grew up alongside smartphones, digital platforms, and constant online connectivity, social media is not simply a communication tool but a social environment in which belonging, visibility, and self-presentation are continuously negotiated [2]. While digital platforms may offer social support, entertainment, peer connection, and access to information, they may also expose young users to social comparison, emotional pressure, excessive engagement, privacy risks, and psychological distress [3]. Recent evidence suggests that the relationship between social media use and mental health is complex and context-dependent, with adverse outcomes more likely to emerge when social media use becomes passive, excessive, emotionally driven, or difficult to regulate [4,5].

One psychological construct that has received increasing attention in this context is fear of missing out (FOMO). FOMO refers to the uncomfortable feeling that others may be having rewarding experiences from which one is absent, often accompanied by a desire to stay continuously connected to what others are doing [6]. In digital environments, this experience may be intensified by real-time updates, curated images, short videos, notifications, and visible indicators of peer activity [7]. FOMO has been linked to problematic social media use, social comparison, lower self-esteem, anxiety, depression, loneliness, and reduced well-being among adolescents and young adults [8]. A recent scoping review also highlighted that FOMO plays a pivotal role in the association between digital media use and mental health, supporting the need to examine FOMO as both a psychological response and a public health concern in highly connected populations [7,8].

Generation Z may be particularly vulnerable to FOMO because social media is closely tied to their peer relationships, identity formation, and sense of social belonging [1]. As digital natives, many individuals in this cohort operate within a blended offline–online environment in which communication, self-expression, and social interaction occur simultaneously across multiple platforms [9]. Within these environments, social media serves as a key space for identity construction, where individuals continuously present themselves and receive social feedback from others. Recent studies among young adults and college students have demonstrated that FOMO is positively associated with problematic smartphone use [10]. Moreover, FOMO has been linked to broader patterns of social media addiction and related behavioral risks among student populations [11]. In addition, FOMO has been shown to contribute to psychological distress, including depressive symptoms and feelings of social exclusion [12]. However, these associations are not uniform across all individuals, as research has identified distinct profiles of FOMO with varying levels of vulnerability and behavioral outcomes [13].

Digital intelligence is another important construct in understanding FOMO among young people, particularly in the context of increasingly complex digital environments [14]. Digital intelligence extends beyond basic digital literacy by encompassing the ability to participate safely, ethically, critically, and responsibly in digital spaces [15]. It includes competencies such as privacy management, digital identity development, critical thinking, screen time regulation, cyberbullying management, cybersecurity awareness, digital footprint monitoring, and digital empathy [15]. From a public health perspective, digital intelligence is crucial because young people must not only access digital platforms but also evaluate information, manage exposure, protect their privacy, regulate online behavior, and maintain psychological well-being [14]. However, the relationship between digital intelligence and FOMO may not be straightforward, as digital environments can simultaneously offer protective and risk-enhancing mechanisms [9]. Higher levels of digital intelligence may promote healthier online behaviors and better self-regulation, reducing problematic engagement [14]. At the same time, greater digital engagement and awareness of online activities may increase exposure to social comparison and perceived social exclusion, which are key drivers of FOMO [9]. Recent research highlights that individual differences in digital experiences and competencies can shape how users respond to social media, including their vulnerability to FOMO-related distress [13].

Self-disclosure is also central to young people’s experiences on social media, reflecting the increasing integration of digital communication into everyday social interaction [16]. Online self-disclosure refers to the sharing of personal information, thoughts, emotions, experiences, images, and opinions through digital platforms [16]. For Generation Z, self-disclosure may serve several functions, including identity expression, relationship maintenance, peer validation, social support, and a sense of belonging [17]. At the same time, self-disclosure may increase vulnerability to privacy concerns, negative evaluation, unwanted attention, and potential misuse of personal information in online environments [18]. In addition, these risks may contribute to social comparison processes and emotional fatigue, particularly in highly connected social media contexts [19]. Prior research among students has shown that FOMO and social media fatigue are closely related phenomena, suggesting that psychological pressures associated with online engagement may shape users’ disclosure behaviors [20]. Other recent studies further indicate that self-presentation and FOMO can operate together as mediating mechanisms linking social media use to problematic or addictive patterns of social media engagement [21].

Although FOMO has been widely studied in relation to social media use and problematic digital behavior, less is known about how emotional intelligence, digital intelligence, and self-disclosure jointly relate to FOMO among Generation Z in Thailand. This gap is important because Thailand represents a collectivist cultural context in which social belonging, peer connection, interpersonal harmony, and online group participation may shape how young people experience and respond to digital social environments. Existing research has often examined FOMO as an outcome of social media use or as a predictor of problematic use, but fewer studies have tested integrated models that include emotional competencies, digital competencies, and online self-disclosure as interrelated factors. Understanding these relationships may help inform public health strategies that move beyond limiting screen time alone and instead promote emotional regulation, digital well-being, privacy awareness, and healthier online communication.

The proposed conceptual framework was informed by the view that FOMO emerges from both individual psychological capacities and social behaviors in digital environments. Emotional intelligence may help individuals recognize and regulate feelings of exclusion, comparison, and anxiety when exposed to others’ online activities. Digital intelligence may shape how young people manage privacy, screen time, digital identity, and social interaction, although greater digital competence may also reflect higher exposure to online social cues. Self-disclosure was positioned as a potential mediating factor because sharing personal information online may reflect how young people seek belonging, validation, and social connection. In this sense, self-disclosure may serve as a behavioral pathway through which emotional and digital competencies are expressed in social media environments and may influence FOMO.

**Figure 1:**
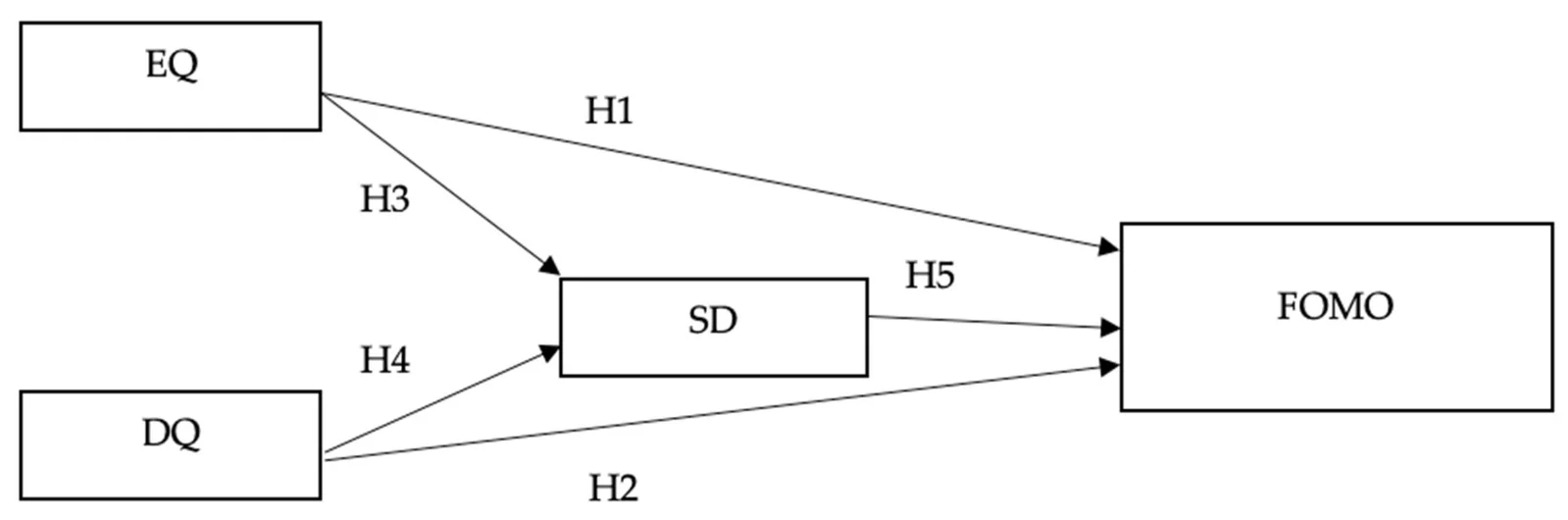
Conceptual framework of emotional intelligence (EQ), digital intelligence (DQ), self-disclosure (SD), and fear of missing out (FOMO).

Therefore, this study aimed to examine the direct and indirect associations among emotional intelligence, digital intelligence, self-disclosure, and FOMO among Generation Z individuals in Thailand. Specifically, we tested whether emotional intelligence and digital intelligence were directly associated with FOMO and whether self-disclosure functioned as a mediating factor in these relationships. Based on the conceptual framework (Figure 1), we proposed the following hypotheses: H1: emotional intelligence is significantly associated with FOMO; H2: digital intelligence is significantly associated with FOMO; H3: emotional intelligence is significantly associated with self-disclosure; H4: digital intelligence is significantly associated with self-disclosure; H5: self-disclosure is significantly associated with FOMO; H6: self-disclosure mediates the association between emotional intelligence and FOMO; and H7: self-disclosure mediates the association between digital intelligence and FOMO. By focusing on psychological and digital competencies within a Generation Z sample, this study contributes to the growing public health literature on digital well-being and youth mental health. The findings may inform future measurement development and confirmatory research on digital wellbeing and youth mental health.

## 2. Materials and Methods

### 2.1. Study Design and Participants

We employed a cross-sectional quantitative research design to examine factors associated with fear of missing out (FOMO) among Generation Z individuals. The target population consisted of young people in Thailand born between 1995 and 2010 who reported active use of social media and digital platforms. A non-probability convenience sampling approach was used to recruit participants. Based on Cochran’s formula [22] for sample size estimation at a 95% confidence level, 400 participants were initially targeted. A total of 400 questionnaires were distributed, and 322 valid responses were retained after data screening for completeness and consistency. This final sample size was considered adequate for PLS-SEM analysis based on the complexity of the proposed model and the number of structural paths examined.

### 2.2. Data Collection Procedure

Data were collected between December 2025 and January 2026 using a structured self-administered questionnaire. Both online and paper-based formats were used to reach participants with different access preferences. The online questionnaire was distributed through social media platforms, whereas the paper-based questionnaire was administered in educational settings. Before completing the questionnaire, participants received information about the study purpose, voluntary participation, confidentiality, and their right to withdraw from the study at any time without penalty. Informed consent was obtained prior to participation. For participants aged below 18 years, written informed consent was obtained from a parent or legal guardian, and assent was obtained from the participant before participation. Participants aged 18 years or older provided their own informed consent. To protect confidentiality, responses were de-identified before analysis, and no names or student identification numbers were entered into the analytic dataset. Paper questionnaires were stored securely, and any information used for recruitment or distribution was kept separately from survey responses. Participants completed the questionnaire within approximately 20–30 min on average. Some participants required up to 45 min because the questionnaire included several multidimensional scales.

### 2.3. Measures

Emotional Intelligence (EQ) was measured using a multidimensional scale adapted from the Thailand Department of Mental Health framework [23], comprising 36 items covering self-regulation, empathy, responsibility, self-motivation, problem-solving, interpersonal relationships, self-esteem, life satisfaction, and post-traumatic growth. Responses were rated on a five-point Likert scale ranging from 1 (strongly disagree) to 5 (strongly agree). Content validity was assessed using the IOC method, with all items achieving acceptable IOC values. The reliability of the EQ scale was high, with a Cronbach’s alpha coefficient of α = 0.93.

Digital Intelligence (DQ) was assessed using the DQ Institute framework [24], which comprises 32 items across eight dimensions: digital identity, screen time management, cyberbullying management, cybersecurity, privacy management, critical thinking, digital footprint awareness, and digital empathy. Responses were rated using a five-point Likert scale ranging from 1 (strongly disagree) to 5 (strongly agree). Content validity was confirmed through the IOC method, with all items exceeding the recommended threshold of 0.96. The reliability of the DQ scale was high, with a Cronbach’s alpha coefficient of α = 0.95.

Self-Disclosure (SD) was measured as a mediating variable reflecting the amount and depth of personal information shared on social media platforms. The items were adapted from prior research on self-disclosure [25] and social penetration theory [26], which had 20 items. Responses were rated on a six-point scale ranging from “absolutely not true” to “absolutely true,” with higher scores indicating greater self-disclosure. Content validity was established using the IOC method, with all values above 0.96. The reliability of the self-disclosure scale was high, with a Cronbach’s alpha coefficient of α = 0.94.

Fear of Missing Out (FOMO) was measured as the dependent variable using a 15-item scale adapted from existing validated instruments [27]. The scale assessed the extent to which individuals experience concern about missing social experiences and the need to remain continuously connected with others. Responses were rated on a five-point Likert scale ranging from 1 (strongly disagree) to 5 (strongly agree). Content validity was verified using the IOC method, with all items achieving acceptable IOC values. The reliability of the FOMO scale was high, with a Cronbach’s alpha coefficient of α = 0.89.

All instruments were reviewed for appropriateness in the Thai context before data collection. The wording of the items was checked to ensure conceptual consistency with the original constructs and clarity for Thai Generation Z participants. Content validity was assessed by experts using the item-objective congruence (IOC) method. Minor wording adjustments were made to improve readability and cultural relevance while preserving the meaning of the original constructs. A pilot reliability assessment was also conducted before the main data collection, and all scales demonstrated acceptable to high internal consistency.

Demographic information collected in this study included gender, age, educational level, marital status, and religion.

### 2.4. Data Analysis

Data were analyzed using descriptive statistics and partial least squares structural equation modeling (PLS-SEM). Frequencies and percentages were used to summarize participant characteristics, whereas means and standard deviations were used to describe the study variables. Before the PLS-SEM analysis, the dataset was screened for missing values, implausible response patterns, data-entry errors, and univariate outliers. The direction and coding of all questionnaire items were also reviewed before scale scores and indicators were entered into the model.

PLS-SEM was conducted using SmartPLS version 4.0. This approach was selected because the proposed model included multiple latent constructs, simultaneous direct and indirect associations, and an exploratory, prediction-oriented objective. The analysis followed a two-stage procedure in which the measurement model was assessed before interpretation of the structural model.

The measurement model was evaluated using indicator loadings, Cronbach’s alpha, rho_A, average variance extracted (AVE), and the heterotrait–monotrait ratio of correlations (HTMT). Outer loadings of approximately 0.70 or higher were considered preferable. Indicators with loadings between 0.40 and 0.70 were reviewed in relation to their theoretical relevance, content coverage, and effects on construct reliability and convergent validity. Indicators were not removed solely to improve statistical criteria when their removal would substantially narrow the conceptual coverage of the multidimensional measures. Internal consistency was considered acceptable when reliability coefficients were at least 0.70. Convergent validity was assessed using AVE, with values of 0.50 or higher considered desirable. Discriminant validity was evaluated using HTMT, with values below 0.85 or 0.90 considered acceptable.

Because the AVE values for the four constructs remained below the conventional 0.50 threshold, convergent validity was considered limited. High internal consistency was not interpreted as compensating for inadequate convergent validity. Consequently, the structural-model findings were treated as preliminary and exploratory rather than as confirmatory estimates.

The structural model was assessed using standardized path coefficients, t-values, *p*-values, coefficients of determination (R^2^), effect sizes (f^2^), and predictive relevance (Q^2^). Direct and indirect effects were evaluated using two-tailed bootstrapping with 5000 resamples. Statistical significance was determined using *p* < 0.05 and 95% bootstrap confidence intervals, with an indirect effect considered statistically significant when its confidence interval did not include zero.

Given the comparatively strong association between digital intelligence and FOMO, this path was interpreted together with the measurement-model results, discriminant-validity assessment, and the possibility of shared digital-context variance or incomplete empirical separation between the constructs. Common method bias was examined using Harman’s single-factor test; however, because the variables were measured using self-report questionnaires during the same data-collection period, potential common method bias and response fatigue were retained as study limitations. Age was analyzed descriptively and was not used for subgroup PLS-SEM analysis because the age groups were unequal in size and the study was not designed or powered for between-group structural comparisons.

## 3. Results

### 3.1. Demographic Characteristics

A total of 322 valid responses were included in the analysis. Slightly more than half of the participants were female (50.30%), and most were aged 19–21 years (53.31%). The largest proportion held a bachelor’s degree (37.27%), while most participants were single (85.72%) and identified as Buddhist (86.02%). These findings indicate that the sample was predominantly young, single, Buddhist, and enrolled in or had completed undergraduate-level education (see Table 1).

### 3.2. Measurement Model Assessment

The measurement model was re-evaluated by examining indicator loadings, internal consistency reliability, convergent validity, and discriminant validity. The observed indicator-loading ranges were 0.426–0.642 for emotional intelligence, 0.522–0.752 for digital intelligence, 0.231–0.838 for self-disclosure, and 0.113–0.810 for FOMO. These ranges show that several indicators, particularly within self-disclosure and FOMO, were weak. No additional indicators were removed during the revision because selective post hoc deletion based solely on statistical performance could reduce the conceptual breadth of the multidimensional scales. Appendix A, Table A1, summarizes the original scale lengths, available loading ranges, and the item-retention decision.

As shown in Table 2, Cronbach’s alpha and rho_A exceeded 0.70 for all constructs, indicating high internal consistency. In contrast, AVE ranged from 0.300 to 0.452 and was below the conventional 0.50 threshold for every construct. Convergent validity was therefore not established. HTMT values in Appendix A, Table A2 were below the commonly used cutoffs; however, this evidence of empirical distinction does not resolve the weak convergent validity. The structural findings are consequently presented as preliminary, exploratory estimates and should not be interpreted as a confirmed measurement or causal model.

### 3.3. Structural Model and Hypothesis Testing

The structural model was evaluated using standardized path coefficients, t-values, and *p*-values. Digital intelligence was positively associated with FOMO (β = 0.717, *p* < 0.001), whereas emotional intelligence was not significantly associated with FOMO (β = −0.065, *p* = 0.233; Table 3). Given the limitations of the measurement model, these estimates should be interpreted as preliminary associations rather than confirmatory effects. H2 was supported within the exploratory model, while H1 was not supported.

### 3.4. Mediation Analysis

**Figure 2:**
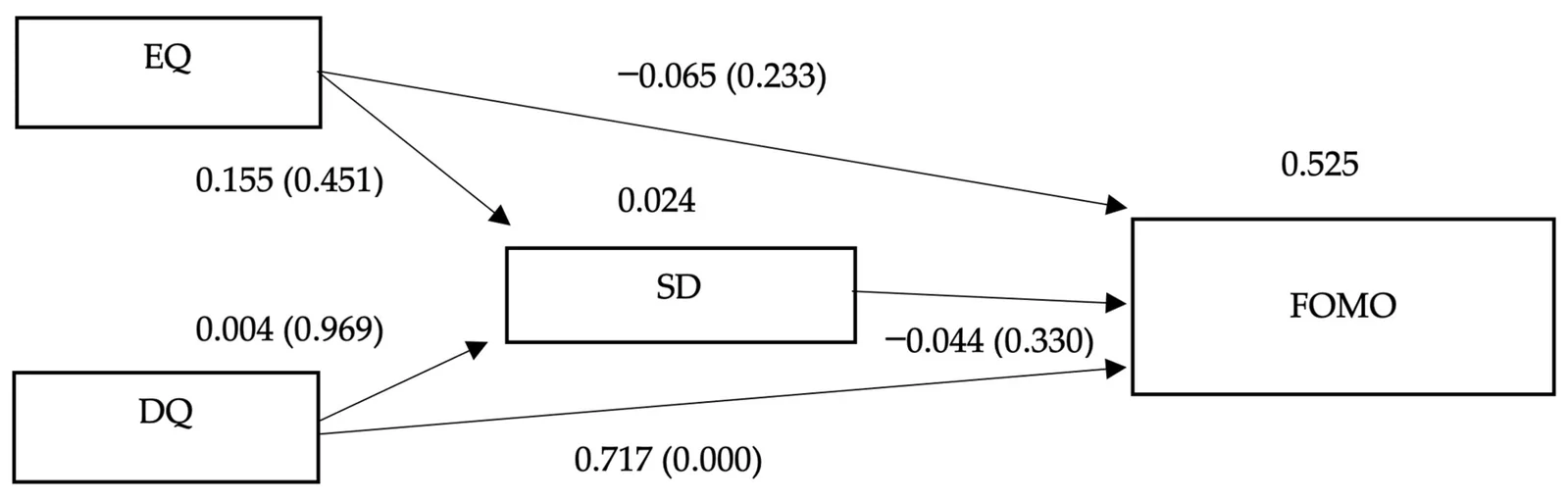
Structural model showing standardized path coefficients, *p*-values, and explained variance for emotional intelligence (EQ), digital intelligence (DQ), self-disclosure (SD), and fear of missing out (FOMO). Note. Values on the arrows represent standardized path coefficients, with *p*-values shown in parentheses. Values above endogenous constructs represent coefficients of determination (R^2^).

Mediation analysis was conducted to examine whether self-disclosure mediated the associations between emotional intelligence, digital intelligence, and FOMO. The results showed that emotional intelligence was not significantly associated with self-disclosure (β = 0.155, *p* = 0.451). Similarly, digital intelligence was not significantly associated with self-disclosure (β = 0.004, *p* = 0.969). Self-disclosure also did not have a significant direct association with FOMO (β = −0.044, *p* = 0.330) (Figure 2).

The indirect effects were also not statistically significant. The indirect effect of emotional intelligence on FOMO through self-disclosure was small and non-significant (β = −0.007, *p* = 0.601). Similarly, the indirect effect of digital intelligence on FOMO through self-disclosure was extremely small and non-significant (β = −0.0002, *p* = 0.977). These findings indicate that self-disclosure did not mediate the associations between emotional intelligence, digital intelligence, and FOMO.

The results of hypothesis testing are summarized in Table 4. Within this exploratory model, digital intelligence was the only construct significantly associated with FOMO. Emotional intelligence and self-disclosure showed no significant direct or indirect associations. These findings remain provisional because convergent validity was not established.

Additional model diagnostics are presented in Appendix A. The model explained 2.4% of the variance in self-disclosure (R^2^ = 0.024) and 52.5% of the variance in FOMO (R^2^ = 0.525). Although the HTMT, f^2^, and Q^2^ results are provided in Appendix A, Table A3, they do not offset the low AVE values; therefore, model performance should be interpreted cautiously.

## 4. Discussion

The present study examined the relationships among Emotional Intelligence (EQ), Digital Intelligence (DQ), self-disclosure (SD), and fear of missing out (FOMO) among Generation Z in Thailand. The findings provide preliminary evidence regarding the relationships among these constructs in a Thai Generation Z sample.

First, the results indicated that Emotional Intelligence (EQ) was not significantly associated with FOMO. This finding contrasts with prior research suggesting that emotional intelligence plays a protective role in adolescent well-being and socioemotional functioning [4,14]. Additional evidence highlights that higher emotional intelligence is generally associated with better psychological adjustment and reduced negative affect among adolescents. However, our results suggest that the protective effect of EQ may be reduced in highly interactive and algorithm-driven digital contexts, where external stimuli such as notifications, curated content, and peer activity continuously demand attention [7,9]. Recent work also indicates that adolescents with higher emotional intelligence may still engage heavily with digital technologies depending on contextual and environmental influences. Thus, even individuals with strong emotional regulation skills may remain vulnerable to FOMO due to persistent exposure to online social comparison and connectivity pressures [28,29].

Second, digital intelligence showed a strong positive association with FOMO (β = 0.717; f^2^ = 0.700). Although these constructs are conceptually distinct, they are both situated within the same digital environment. Digital intelligence refers to the competencies needed to engage with digital technologies safely, critically, and responsibly, whereas FOMO reflects concern that others may be having rewarding experiences elsewhere and a desire to remain continuously connected. The magnitude of the observed path may therefore represent a genuine relationship, shared variance arising from the common digital context, incomplete empirical separation between the measures, or a combination of these factors. Although the HTMT value for digital intelligence and FOMO was below the conventional threshold, this finding does not resolve the concerns created by the low AVE values. Accordingly, the association should be interpreted as a preliminary finding that requires replication with more psychometrically robust measures rather than as a stable estimate.

One possible substantive explanation is that individuals with greater digital competence may also be more active, attentive, and frequent users of digital platforms. This greater engagement could increase exposure to real-time updates, peer activities, and opportunities for social comparison [8,9]. However, the present study cannot clearly distinguish this explanation from the limitations of the measurement model. Future research should examine the dimensions of digital intelligence separately, test alternative measurement structures, and determine whether the association persists after accounting for the intensity, frequency, and pattern of digital-platform use.

From a broader perspective, these findings illustrate a “digital intelligence paradox”: although digital skills are designed to enable safe and responsible technology use [14,15], increased engagement may also expose individuals to heightened psychological risks. This is consistent with recent public health reports emphasizing that digital technologies simultaneously provide benefits (connection, self-expression) and risks (comparison, overexposure, mental health concerns) for young people. Therefore, the role of digital intelligence should be reconsidered not only as a protective factor but also as a potential amplifier of digital exposure [30].

Third, self-disclosure (SD) did not have a significant direct effect on FOMO and did not mediate the relationships between EQ, DQ, and FOMO. Although self-disclosure is widely recognized as a central mechanism for identity development and relationship building in digital environments [16,17], it also involves complex trade-offs. On one hand, self-disclosure can enhance social capital, belonging, and interpersonal trust. On the other hand, it may increase vulnerability to privacy risks, social evaluation, and emotional fatigue [18,19]. Recent studies also indicate that online self-disclosure behaviors may be influenced by platform design, privacy concerns, and perceived social norms, which can complicate their effects on psychological outcomes [31,32].

Moreover, research suggests that FOMO is more strongly related to passive social media consumption and social comparison rather than active content sharing. This helps explain why self-disclosure did not emerge as a significant predictor in our model. Additionally, FOMO has been shown to act as a driver of social media fatigue and compulsive engagement, indicating that it may function more as an outcome of exposure rather than disclosure behavior [33,34].

Fourth, the mediation analysis revealed that self-disclosure did not function as a pathway linking EQ or DQ to FOMO. These findings challenge assumptions that self-presentation and disclosure are primary mechanisms through which social media influences psychological outcomes [21]. Instead, recent studies suggest that exposure-related processes—such as constant monitoring of others’ activities, algorithmic amplification, and perceived social exclusion—play a more central role in FOMO development. Furthermore, large-scale studies of Generation Z highlight that technology-related factors, including screen time and continuous information updates, are key contributors to FOMO and reduced well-being [35,36].

These non-significant findings and the strong digital intelligence–FOMO path must be interpreted in light of the measurement limitations. High internal consistency does not by itself demonstrate construct validity, and the low AVE values indicate that substantial indicator variance was not captured by the intended constructs. In addition, several indicators showed suboptimal loadings, and further post hoc deletion was not undertaken because extensive item removal could have reduced the conceptual coverage of the multidimensional measures. The results are therefore exploratory and hypothesis-generating. Future studies should use shorter or more clearly dimensional instruments, report all item-level loadings and deletion decisions, and confirm the measurement model before evaluating structural relationships.

Overall, the findings underscore the complexity of digital well-being among Generation Z. Social media engagement is deeply embedded in this generation’s daily lives, shaping identity, communication, and social dynamics [1,2]. However, increasing evidence suggests that the mental health effects of social media are not uniform but depend on patterns of use, individual differences, and contextual factors [4,5]. For example, high-frequency use, exposure to idealized content, and comparison-oriented behaviors are consistently associated with poorer mental health outcomes. Taken together, our results align with a growing body of literature emphasizing that FOMO reflects a broader interaction between individual psychological factors and external digital environments. Rather than being driven by a single factor, FOMO appears to emerge from a combination of social comparison, connectivity pressures, digital exposure, and perceived social exclusion.

### 4.1. Implications for Public Health and Practice

From a public health perspective, the study identifies questions that warrant further investigation rather than providing a direct basis for intervention or policy. The preliminary results suggest that digital competence and FOMO may be linked in ways that are not captured by technical literacy alone. Before programs are designed around this relationship, future studies should refine the measures, replicate the model, and examine specific forms of digital engagement, social comparison, and emotional regulation. At present, the findings are most useful for guiding instrument development and confirmatory research.

### 4.2. Strengths and Limitations

This study integrates emotional, digital, and behavioral constructs within one model, but several limitations substantially restrict interpretation. First, the cross-sectional design does not establish directionality or causality. Second, AVE was below 0.50 for all constructs, indicating that convergent validity was not established. Third, several indicators showed suboptimal loadings, and no additional items were removed during the revision because extensive post hoc deletion could have reduced the conceptual coverage of the multidimensional measures. Fourth, the strong digital intelligence–FOMO path may partly reflect construct overlap or shared digital-context measurement. Fifth, the lengthy self-report questionnaire may have introduced response fatigue and common method bias. Sixth, convenience sampling limits generalizability, and age-group differences were not examined because group sizes were unequal. These limitations mean that the structural results should be considered preliminary and exploratory until replicated with a fully validated measurement model.

## 5. Conclusions

This study examined the associations among emotional intelligence, digital intelligence, self-disclosure, and fear of missing out among Generation Z individuals in Thailand. Digital intelligence showed a strong positive association with FOMO, whereas emotional intelligence and self-disclosure were not significantly associated with FOMO. Self-disclosure also did not mediate the associations between emotional intelligence, digital intelligence, and FOMO. These findings should be interpreted cautiously. Although the constructs demonstrated acceptable internal consistency, the AVE values were below the conventional threshold, indicating limited convergent validity. The strong association between digital intelligence and FOMO may reflect a substantive relationship, shared variance arising from the common digital context, incomplete empirical separation of the measures, or a combination of these explanations. Accordingly, the results should be regarded as preliminary and hypothesis-generating rather than as stable estimates that can directly inform public health practice or policy. The study nevertheless highlights the need to examine digital wellbeing among young people using measures that clearly distinguish digital competence from psychological responses to online engagement. Future research should refine the measurement model, report item-level performance, examine the dimensions of digital intelligence separately, and account for the frequency, intensity, and pattern of social media use. Longitudinal and mixed-methods studies are also needed to determine whether the observed associations persist over time and to clarify how digital competence, social comparison, and FOMO interact in everyday digital life.

## Figures and Tables

**Table 1:** Demographic characteristics of the participants.

Demographic Characteristics	*n* (%)
Gender	
Male	140 (43.50)
Female	162 (50.30)
LGBTQ+	20 (6.20)
Age	
16–18	43 (13.48)
19–21	172 (53.31)
22–24	107 (33.21)
Education Level	
Lower Secondary School	46 (14.29)
Upper Secondary School/Vocational Certificate	65 (20.19)
Diploma/Higher Vocational Certificate	75 (23.29)
Bachelor’s Degree	120 (37.27)
Master’s Degree	16 (4.96)
Marital Status	
Single	276 (85.72)
Married	42 (13.04)
Separated/Divorced/Widowed	4 (1.24)
Religion	
Buddhism	277 (86.02)
Christianity	40 (12.42)
Islam	5 (1.56)

**Table 2:** Construct-Level Reliability and Convergent Validity.

Construct	Cronbach’s Alpha	Rho_A	AVE
Emotional Intelligence (EQ)	0.934	0.927	0.300
Digital Intelligence (DQ)	0.953	0.956	0.407
Self-disclosure (SD)	0.937	0.899	0.452
Fear of Missing Out (FOMO)	0.900	0.922	0.448

Note: EQ = Emotional Intelligence; DQ = Digital Intelligence; SD = Self-Disclosure; FOMO = Fear of Missing Out in social media. AVE = average variance extracted.

**Table 3:** Significance testing results of the direct effects of EQ and DQ on FOMO.

Hypothesis	Path	Path Coefficients	STDEV	t-Value	*p*-Value	Result
H1	EQ → FOMO	−0.065	0.054	1.193	0.233	Not Supported
H2	DQ → FOMO	0.717	0.034	21.167	<0.001	Supported

Note: EQ = Emotional Intelligence; DQ = Digital Intelligence; FOMO = Fear of Missing Out in social media.

**Table 4:** Summary of Hypothesis Testing.

Hypothesis	Path	Standardized Estimate	t-Value	*p*-Value	Result
H1	EQ → FOMO	−0.065	1.193	0.233	Not Supported
H2	DQ → FOMO	0.717	21.167	<0.001	Supported
H3	EQ → SD	0.155	0.754	0.451	Not Supported
H4	DQ → SD	0.004	0.039	0.969	Not Supported
H5	SD → FOMO	−0.044	0.974	0.330	Not Supported
H6	EQ → SD → FOMO	-0.007	0.522	0.601	Not Supported
H7	DQ → SD → FOMO	−0.0002	0.029	0.977	Not Supported

Note: EQ = Emotional Intelligence; DQ = Digital Intelligence; SD = Self-Disclosure; FOMO = Fear of Missing Out in social media.

## Data Availability

The data presented in this study are available from the corresponding author upon reasonable request. The data are not publicly available due to privacy and ethical restrictions related to participant confidentiality.

## Abbreviations

The following abbreviations are used in this manuscript:

DQ	Digital Intelligence
EQ	Emotional Intelligence
FOMO	Fear of Missing Out
PLS-SEM	Partial Least Squares Structural Equation Modeling
SD	Self-Disclosure
SEM	Structural Equation Modeling
SNSs	Social Networking Sites

## Appendix A

### Appendix A.1. Measurement Inventory and Item-Retention Summary

**Table A1:** Measurement Inventory and Item-Retention Decisions.

Construct	Original Items	Observed Loading Range	Additional Items Removed During Revision	Construct-Level Reliability and Validity
EQ	36	0.426–0.642	None	α = 0.934; rho_A = 0.927; AVE = 0.300
DQ	32	0.522–0.752	None	α = 0.953; rho_A = 0.956; AVE = 0.407
SD	20	0.231–0.838	None	α = 0.937; rho_A = 0.899; AVE = 0.452
FOMO	15	0.113–0.810	None	α = 0.900; rho_A = 0.922; AVE = 0.448

Note. No additional indicators were removed during the present revision because item deletion based solely on statistical performance could have reduced the conceptual coverage of the multidimensional measures. The table reports the observed indicator-loading ranges and construct-level reliability and validity results. Because the AVE values remained below the conventional threshold of 0.50, convergent validity was not established, and the structural findings were interpreted as preliminary and exploratory.

### Appendix A.2. Discriminant Validity Assessment Using HTMT

**Table A2:** Discriminant validity assessment using the heterotrait–monotrait ratio of correlations (HTMT).

Construct	EQ	DQ	SD	FOMO
EQ	-			
DQ	0.62	-		
SD	0.55	0.48	-	
FOMO	0.41	0.78	0.39	-

Note. EQ = Emotional Intelligence; DQ = Digital Intelligence; SD = Self-Disclosure; FOMO = Fear of Missing Out. HTMT values below 0.85 or 0.90 indicate acceptable discriminant validity.

### Appendix A.3. Structural Model Quality Indicators

**Table A3:** Structural model quality indicators.

Endogenous Construct	R^2^	Q^2^	Predictor	f^2^	Interpretation
FOMO	0.525	0.210	EQ	0.010	Very small
FOMO	0.525	0.210	DQ	0.700	Large
FOMO	0.525	0.210	SD	0.003	Very small
SD	0.024	0.006	EQ	0.020	Small
SD	0.024	0.006	DQ	0.000	Very small

Note. R^2^ = coefficient of determination; Q^2^ = predictive relevance; f^2^ = effect size. R^2^ values of 0.25, 0.50, and 0.75 may be interpreted as weak, moderate, and substantial explanatory power, respectively. Q^2^ values greater than 0 indicate predictive relevance. f^2^ values of 0.02, 0.15, and 0.35 indicate small, medium, and large effect sizes, respectively.

## References

[1] Pérez-Torres V. (2024). Social media: A digital social mirror for identity development during adolescence. Curr. Psychol..

[2] Öksüz Y.E., Çetin F. (2026). Digital Identity and Ideal Self-Presentation on Social Media: A Generation Z Study within the Society of the Spectacle. Kronotop İletişim Derg..

[3] Ndindeng A. (2025). The impact of social media on mental health. Ment. Health Digit. Technol..

[4] Agyapong-Opoku N., Agyapong-Opoku F., Greenshaw A.J. (2025). Effects of Social Media Use on Youth and Adolescent Mental Health: A Scoping Review of Reviews. Behav. Sci..

[5] Odgers C.L., Jensen M.R. (2020). Annual Research Review: Adolescent mental health in the digital age: Facts, fears, and future directions. J. Child. Psychol. Psychiatry.

[6] Gupta M., Sharma A. (2021). Fear of missing out: A brief overview of origin, theoretical underpinnings and relationship with mental health. World J. Clin. Cases.

[7] Tukur M., Schneider J., Househ M., Dokoro A.H., Ismail U.I., Dawaki M., Agus M. (2024). The Metaverse digital environments: A scoping review of the techniques, technologies, and applications. J. King Saud Univ.—Comput. Inf. Sci..

[8] Groenestein E., Willemsen L., van Koningsbruggen G.M., Ket H., Kerkhof P. (2024). The relationship between fear of missing out, digital technology use, and psychological well-being: A scoping review of conceptual and empirical issues. PLoS ONE.

[9] Nesbit T., Lole L. (2026). A Qualitative Investigation of Social Media Fear of Missing Out (FoMO) and Needs Fulfilment Among Young Adults. Int. J. Hum.–Comput. Interact..

[10] Guan J., Ma W., Liu C. (2023). Fear of missing out and problematic smartphone use among Chinese college students: The roles of positive and negative metacognitions about smartphone use and optimism. PLoS ONE.

[11] Jing Z., Yang W., Lei Z., Junmei W., Hui L., Tianmin Z. (2025). Correlations between social media addiction and anxiety, depression, FoMO, loneliness and self-esteem among students: A systematic review and meta-analysis. PLoS ONE.

[12] Gao B., Shen Q., Luo G., Xu Y. (2023). Why mobile social media-related fear of missing out promotes depressive symptoms? the roles of phubbing and social exclusion. BMC Psychol..

[13] Li J., Zhou Y., Liu Y., Yu Z., Gao X. (2024). Profiles of fear of missing out and their social media use among young adults: A six-month longitudinal study. Addctv. Behav..

[14] Salazar I.C., Santamaría-Perales R., Cuevas-Toro A.M. (2025). Are FoMO, Experiential Avoidance, and Emotional Distress Related to Problematic Social Network Use in Young Adults?. Healthcare.

[15] Tan Q. (2025). The Impact of Social Media Use on Adolescent Mental Health: The Mediating Role of FOMO. Commun. Humanit. Res..

[16] Bai Q., Dan Q., Choi Y., Luo S. (2025). Why We Disclose on Social Media? Towards a Dual-Pathway Model. Behav. Sci..

[17] Șurariu C., Carnelley K.B., Hart C.M. (2025). The social benefits of self-disclosure and self-presentation through social media: A systematic review. Behav. Inf. Technol..

[18] Sun J., Ghazali A.H.A., Abdul R.S.N. (2025). Risky online self-disclosure in adolescents: A meta-analytic review of predictors and outcomes. Front. Psychol..

[19] Yao X., Zhao J., Chao W., Gao D., Wang M., Zhao G. (2025). Relationship Between Fear of Missing Out and Social Media Fatigue: Cross-Lagged Panel Design. J. Med. Internet Res..

[20] Jabeen F., Tandon A., Sithipolvanichgul J., Srivastava S., Dhir A. (2023). Social media-induced fear of missing out (FoMO) and social media fatigue: The role of narcissism, comparison and disclosure. J. Bus. Res..

[21] Ho W.W.Y., Lau Y.H.Y., Leung L.Y.L., Li E.K.L., Ma R.K.K. (2024). Enigma of social media use: Complexities of social media addiction through the serial mediating effects of emotions and self-presentation. Front. Psychol..

[22] Cochran W.G., Carroll S.P. (1953). A Sampling Investigation of the Efficiency of Weighting Inversely as the Estimated Variance. Biometrics.

[23] Department of Mental Health Ministry of Public Health EQ Manual: Emotional Intelligence. Mental Health Knowledge Repository, Small Book Edition. https://dmh-elibrary.org/items/show/1199.

[24] Park Y. DQ Global Standards Report. https://www.dqinstitute.org/wp-content/uploads/2019/11/DQGlobalStandardsReport2019.pdf.

[25] Jourard S.M. (1971). Self-Disclosure: An Experimental Analysis of the Transparent Self.

[26] Altman I., Taylor D.A. (1973). Social Penetration: The Development of Interpersonal Relationships.

[27] Chancharoen R., Aphisamacharayotin P. (2018). The relationship between social media using and fear of mission out of university stu-dents in Phitsanulok province. Human. Resour. Organ. Dev. J..

[28] Llamas-Díaz D., Cabello R., Gómez-Leal R., Gutiérrez-Cobo M.J., Megías-Robles A., Fernández-Berrocal P. (2023). Ability Emotional Intelligence and Subjective Happiness in Adolescents: The Role of Positive and Negative Affect. J. Intell..

[29] Piombo M.A., La Grutta S., Epifanio M.S., Di Napoli G., Novara C. (2025). Emotional Intelligence and Adolescents’ Use of Artificial Intelligence: A Parent–Adolescent Study. Behav. Sci..

[30] Chhabra J., Pilkington V., Benakovic R., Wilson M.J., La Sala L., Seidler Z. (2025). Social media and youth mental health: Scoping review of platform and policy recommendations. J. Med. Internet Res..

[31] Gavriilidou O., Gritzalis S. (2025). Unmasking the True Self on Social Networking Sites. Psychol. Int..

[32] Wang W., Wu Q., Li D., Tian X. (2025). An exploration of the influencing factors of privacy fatigue among mobile social media users from the configuration perspective. Sci. Rep..

[33] Huang X. (2025). Mindfulness in social media exposure: The pressure-reducing valve for fear of missing out and social media fatigue. Front. Psychol..

[34] Castillo-Gualda R., Rathje S., Ramos-Cejudo J. (2026). Social comparison and maladaptive emotion regulation are associated with poorer mental health in social media users. Sci. Rep..

[35] Dwidienawati D., Pradipto Y., Indrawati L., Gandasari D. (2025). Internal and external factors influencing Gen Z wellbeing. BMC Public Health.

[36] Littman-Ovadia H., Russo-Netzer P. (2024). Exploring the lived experience and coping strategies of Fear of Missing Out (FoMO) among emerging adults. Curr. Psychol..

